# Assessment of Survivor Concerns (ASC): A newly proposed brief questionnaire

**DOI:** 10.1186/1477-7525-5-15

**Published:** 2007-03-13

**Authors:** Carolyn C Gotay, Ian S Pagano

**Affiliations:** 1 Cancer Research Center of Hawaii, Honolulu, HI 96813, USA

## Abstract

**Background:**

The purpose of this study was to design a brief questionnaire to measure fears about recurrence and health in cancer survivors. Research involving fear of recurrence has been increasing, indicating that it is an important concern among cancer survivors.

**Methods:**

We developed and tested a six-item instrument, the Assessment of Survivor Concerns (ASC). Construct validity was examined in a multiple group confirmatory factor analysis (CFA) with 592 short-term and 161 long-term cancer survivors. Convergent and discriminant validity was examined through comparisons with the PANAS (Positive and Negative Affect Schedule) and the CES-D (Center for Epidemiologic Studies Depression) measures.

**Results:**

CFA models for the ASC with short- and long-term survivors showed good fit, with equivalent structure across both groups of cancer survivors. Convergent and discriminant validity was also supported through analyses of the PANAS and CES-D. One item (children's health worry) did not perform as well as the others, so the models were re-run with the item excluded, and the overall fit was improved.

**Conclusion:**

The ASC showed excellent internal consistency and validity. We recommend the revised five-item instrument as an appropriate measure for assessment of cancer survivor worries.

## Background

As treatments and detection for cancer improve, life expectancy for cancer survivors is longer than it has ever been before. While this is of course a welcome development, there are potential concerns that need to be addressed. One of these is the constant fear experienced by many cancer survivors that their cancer will return. While the risk of recurrence varies considerably depending on the cancer type, tumor characteristics, and stage at diagnosis, many cancers do return, generally in the first few years following diagnosis, but sometimes even after many years of remission. *Fear of cancer recurrence *is recognized as having significant negative psychological consequences, and researchers have recently taken a greater interest in the construct. Lee-Jones et al. provided an important review of the work that had been done in this area up to 1997 [1].

Northouse reported the first work on the development of a scale specific for measuring fear of cancer recurrence, the 22-item Northouse Fear of Recurrence Scale [2]. The scale was developed for the purpose of testing a hypothesized relationship between the availability of significant others and reduced fear of recurrence. Northouse reported that the scale had adequate reliability, and later studies using the scale also found adequate reliability, but validity assessment was not performed [3,4].

Other early measures of fear of cancer recurrence include the Worry about Cancer Scale [5] and the Fear of Recurrence Index [5,6]. The Worry about Cancer Scale was based on cognitive-behavioral theory, and focused on perceptual cues that could elicit worry. The Fear of Recurrence Index was developed to test the relation between type of surgery and fear of recurrence.

More recently, Vickberg (2003) developed the 29-item Concerns About Recurrence Scale (CARS) [7], which assesses the extent and nature of women's fears about the possibility of breast cancer recurrence. The CARS was found to be internally consistent, and exploratory factor analysis revealed four worry factors: health, womanhood, role, and death.

As part of a more comprehensive study assessing quality of life in adult long-term cancer survivors, Avis et al. (2005) developed the 47-item Quality of Life in Adult Cancer Survivors (QLACS) [8]. The participants in this study were all five years or more post-diagnosis. Through exploratory factor analysis, the QLACS was shown to be a multi-dimensional instrument with 12 subscales. One of the subscales is characterized as "recurrence distress" and contains four items related to fear of recurrence. Another is characterized as "family distress" and contains three items related to fear of family members getting cancer. Both subscales showed adequate reliability and criterion-based validity. These data suggest that fear of cancer recurrence is a distinct and important construct that should be studied further.

The purpose of this study was to develop and evaluate a brief (only six items) instrument, which is specific to two factors: fear of cancer recurrence and fear of health issues in general. It was not intended to assess overall quality of life, but rather to serve as an adjunct – or module – to other quality of life questionnaires. Also, in contrast to previous research, which has used exploratory analyses, we tested a theoretical model of fear of recurrence through confirmatory factor analysis. Further, because fear of cancer recurrence has the potential to be defined differently between survivors who are at different points post-diagnosis, we included both long-term (5–6 years post-diagnosis) and short-term (1.5–2.5 years post-diagnosis) cancer survivors in this study.

Based on interviews and comments on open-ended questions in previous research with cancer survivors, we developed six items for the instrument (see Table 1), which was named the Assessment of Survivor Concerns (ASC). Three items (recurrence, new diagnosis, diagnostic tests) were specific to cancer worry (forming a cancer worry subscale), and three (death, health, and children's health) assessed general health worry (forming a health worry subscale).

**Table 1:** Cancer Worry Questionnaire Correlations

Item	I worry about...	Subscale	1	2	3	4	5	6
1	future diagnostic tests	Cancer Worry	1.00					
2	another type of cancer	Cancer Worry	0.81	1.00				
3	my cancer coming back	Cancer Worry	0.76	0.87	1.00			

4	dying	Health Worry	0.33	0.31	0.28	1.00		
5	my health	Health Worry	0.34	0.35	0.33	0.58	1.00	
6	my children's health	Health Worry	0.31	0.31	0.30	0.27	0.38	1.00

Each item had a Likert-type scale with four response options: 1 = Not at all; 2 = A little bit; 3 = Somewhat; 4 = Very much. All correlations are statistically significant at the 0.0001-level.

In an assessment of construct validity, the cancer specific and the general health constructs were hypothesized to be distinct, but with variance in common. Additionally, because worry is a form of distress, both constructs were hypothesized to be related to, but distinct from, other negative affect constructs (e.g., depression). In an assessment of convergent validity, we predicted that cancer and health worry would be significantly correlated with negative affect measures. We hypothesized that this correlation should not be very large in magnitude since the cancer and health worry constructs were seen as distinct from other negative affect constructs. Also, in an assessment of discriminant validity, we predicted that cancer and health worry would have small or zero correlations with measures of positive affect. If both convergent and discriminant validity were found, this would provide further evidence in support of construct validity.

In this paper, when we refer to all six items as a whole, we will use the qualifiers *questionnaire *or *instrument*. When referring to either the three cancer specific items or to the three general health items, we will use the qualifier *subscale*. When referring to a particular item, we will use the qualifier *item*. Additionally, we use the terms *construct *and *factor *interchangeably when referring to the latent constructs (e.g., cancer worry and health worry) that are assessed by the observed items.

## Methods

### Participants

Cancer survivors were identified through the population-based Hawaii Tumor Registry (HTR), a member of the National Cancer Institute-supported Surveillance, Epidemiology, and End Results (SEER) Registry, which maintains records for all cancers diagnosed in the state. Eligibility criteria were histologic confirmation of cancer diagnosed 1.5–2.5 years prior to assessment (short-term survivors) or 5–6 years prior to assessment (long-term survivors), localized stage of disease, cancer-free status at time of assessment, ability to understand English, permission of primary physician, Hawaii residency, and at least 18 years of age.

There were 1,323 survivors identified who met the eligibility criteria. These individuals were mailed a questionnaire packet, and the items reported in this paper were among a number of scales of quality of life and well-being. Institutional Review Board approval was obtained. We did not obtain written informed consent. The elements of informed consent were included in a covering letter, and return of the survey indicated consent. A total of 753 patients returned questionnaires for an overall response rate of 57%. Response rates were comparable for the 161 long-term (54%) and 592 short-term (58%) survivors. See Table 2 for demographic and clinical characteristics by response status. See Table 3 for demographic and clinical characteristics of the final sample by survivor status.

**Table 2:** Demographic and clinical characteristics by response status

	Responders		Non-responders		Total	
	*n*	*%*	*n*	*%*	*n*	*%*

**Age**						
Under 50	117	15.5	116	20.4	233	17.6
50–65	293	38.9	200	35.1	493	37.3
Over 65	343	45.6	254	44.6	597	45.1

**Sex**						
Female	494	65.6	360	63.2	854	64.6
Male	259	34.4	210	36.8	469	35.4

Marital Status*						
With Partner	524	69.6	346	60.7	870	65.8
No Partner	221	29.3	219	38.4	440	33.3

Ethnicity*						
Japanese	321	42.6	213	37.4	534	40.4
Caucasian	248	32.9	153	26.8	401	30.3
Hawaiian	100	13.3	112	19.6	212	16.0
Filipino	79	10.5	84	14.7	163	12.3
Other	5	0.7	9	1.6	14	1.1

**Treatment**						
Surgery	688	91.4	522	91.6	1210	91.5
Chemotherapy	92	12.2	60	10.5	152	11.5
Radiation	300	39.8	192	33.7	492	37.2
Hormone	160	21.2	95	16.7	255	19.3

Site*						
Breast	296	39.3	173	30.4	469	35.4
Digestive	108	14.3	83	14.6	191	14.4
Prostate	101	13.4	69	12.1	170	12.8
Other	248	32.9	245	43.0	493	37.3

**Total**	753	100.0	570	100.0	1323	100.0

For *Treatment*, responses are not mutually exclusive. Statistically significant differences (*p *< .01) are indicated with an asterisk (*).

**Table 3:** Demographic and clinical characteristics by survivor status

	Short-Term Survivors		Long-Term Survivors		Total Sample	
	*n*	*%*	*n*	*%*	*n*	*%*

Age*						
Under 50	87	14.7	30	18.6	117	15.5
50–65	209	35.3	84	52.2	293	38.9
Over 65	296	50.0	47	29.2	343	45.6

Sex*						
Female	372	62.8	122	75.8	494	65.6
Male	220	37.2	39	24.2	259	34.4

**Marital Status**						
With Partner	413	69.8	100	62.1	513	68.1
No Partner	179	30.7	61	37.9	240	31.2

**Ethnicity**						
Japanese	233	39.4	74	46.0	307	40.8
Caucasian	198	33.5	45	28.0	243	32.3
Hawaiian	70	11.8	19	11.8	89	11.8
Filipino	59	10.0	14	8.7	73	9.7
Other	32	5.4	9	5.6	32	5.4

**Children**						
None	98	16.6	27	16.8	125	16.6
One	78	13.2	19	11.8	97	12.9
Two	160	27.0	52	32.3	212	28.2
Three	98	16.6	30	18.6	128	17.0
Four or More	156	26.4	30	18.6	186	24.7

**Education**						
No Degree	69	11.7	11	6.9	80	10.7
High School	131	22.2	29	18.2	160	21.4
Some College	193	32.8	56	35.2	249	33.3
College Degree	127	21.6	34	21.4	161	21.5
Graduate Degree	69	11.7	29	18.2	98	13.1

**Family Income**						
Under $25,000	173	31.4	45	28.7	218	30.8
$25,000–$50,000	174	31.6	42	26.8	216	30.5
Over $50,000	204	37.0	70	44.6	274	38.7

**Treatment**						
Surgery	465	78.6	133	82.6	598	79.4
Chemotherapy*	82	13.9	39	24.2	121	16.1
Radiation*	262	44.3	87	54.0	349	46.4
Hormone	96	16.2	25	15.5	121	16.1

Site*						
Breast	222	37.5	78	48.4	300	39.8
Digestive	92	15.5	15	9.3	107	14.2
Prostate	84	14.2	16	9.9	100	13.3
Female Genital	59	10.0	19	11.8	78	10.4
Other	135	22.8	33	20.5	168	22.3

**Total**	592	78.6	161	21.4	753	100.0

Only data from the final sample are shown. For *Treatment*, responses are not mutually exclusive. Statistically significant differences (*p *< .01) are indicated with an asterisk (*).

### Measures

The primary measure was the newly developed ASC questionnaire, consisting of six items. These items were developed based on comments from cancer survivors in previous studies of quality of life, in which we asked what was missing in available questionnaires; cancer recurrence and health fears were frequent responses. Candidate items were pretested in cancer survivors in support groups and semi-structured interviews (approximately 20 individuals), who reported that all six candidate items were relevant and clearly phrased. The instrument was divided into two subscales with three items each. The first subscale's items were specific to cancer worry (recurrence, new diagnosis, and diagnostic tests), and the second subscale's items were related to general health worry (death, health, and children's health). See Table 1 for a list of all the questionnaire items along with inter-item correlations, and see Table 4 for the means, standard deviations, and distributions of responses.

**Table 4:** Cancer Worry Questionnaire Response Distributions, Means, and Standard Deviations by Survivor Status

	**Cancer Worry Subscale**						**Health Worry Subscale**					
	Future Tests		New Cancer		Recurrence		Death		Health		Children's Health	

	*n*	*%*	*n*	*%*	*N*	*%*	*n*	*%*	*n*	*%*	*n*	*%*

**Short-term**												
1-Not at all	169	28.5	148	25.0	163	27.5	418	70.6	290	49.0	313	52.9
2-A little bit	103	17.4	107	18.1	110	18.6	116	19.6	194	32.8	105	17.7
3-Somewhat	108	18.2	107	18.1	102	17.2	28	4.7	75	12.7	37	6.3
4-Very much	186	31.4	201	34.0	196	33.1	13	2.2	19	3.2	24	4.1
No Response	26	4.4	29	4.9	21	3.5	17	2.9	14	2.4	113	19.1
Mean/S.D.	2.55	1.20	2.64	1.18	2.58	1.19	1.37	0.68	1.69	0.82	1.52	0.84

**Long-term**												
1-Not at all	38	23.6	32	19.9	39	24.2	116	72.0	70	43.5	72	44.7
2-A little bit	33	20.5	32	19.9	30	18.6	30	18.6	55	34.2	32	19.9
3-Somewhat	39	24.2	36	22.4	34	21.1	7	4.3	23	14.3	25	15.5
4-Very much	47	29.2	57	35.4	57	35.4	5	3.1	12	7.5	5	3.1
No Response	4	2.5	4	2.5	1	0.6	3	1.9	1	0.6	27	16.8
Mean/S.D.	2.61	1.11	2.75	1.12	2.69	1.16	1.37	0.72	1.86	0.93	1.72	0.90

**Total Sample**												
1-Not at all	207	27.5	180	23.9	201	26.7	534	70.9	360	47.8	385	51.1
2-A little bit	136	18.1	139	18.5	140	18.6	146	19.4	249	33.1	137	18.2
3-Somewhat	148	19.7	143	19.0	137	18.2	35	4.6	98	13.0	62	8.2
4-Very much	232	30.8	258	34.3	253	33.6	18	2.4	31	4.1	29	3.9
No Response	30	4.0	33	4.4	22	2.9	20	2.7	15	2.0	140	18.6
Mean/S.D.	2.56	1.18	2.67	1.17	2.60	1.19	1.37	0.69	1.73	0.85	1.57	0.86

For the response distributions for each item, the number of people (*n*) is shown on the left-hand side and the percentage (*%*) on the right. For the means and standard deviations (S.D.), the mean is on the left and the standard deviation is on the right.

Two instruments were used for the assessment of convergent and discriminant validity for the cancer and health worry subscales. The first was the Positive and Negative Affect Schedule (PANAS), which assesses both pleasurable and distressful mood states [9]. The original PANAS instrument consisted of 20 items, but we only included 10. These 10 items have been tested psychometrically, shown to be useful in previous research [10], and previously used with cancer patient populations [11,12]. Five of these 10 items (excited, enthusiastic, determined, alert, and inspired) reflect positive affect, and five (upset, distressed, nervous, scared, and afraid) reflect negative affect.

The second instrument was the Center for Epidemiologic Studies Depression (CESD) scale, which has been used extensively in both community and patient populations, including cancer patients [13-15]. It includes 20 symptom-related four-point items, in which respondents rate the frequency of having experienced these symptoms during the past week. Sixteen of the items are worded to reflect negative affect (depression), and four (felt good, future hopeful, happy, enjoyed life) are worded to reflect positive affect.

### Data analysis

**Figure 1:**
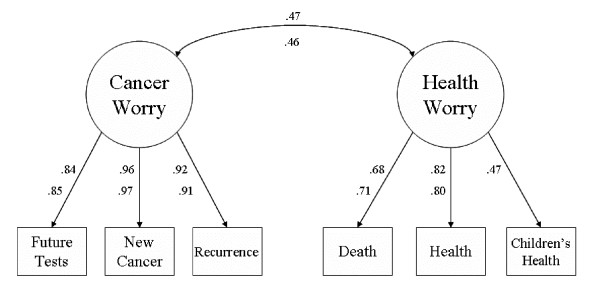
**Confirmatory Factor Models for the Six-Item and Five-Item ASC Instruments**. *Note*. All values are standardized. The numbers on the top are for the original six-item ASC; and the numbers on the bottom are for the revised five-item ASC (children's health item excluded).

Assessment of the construct validity of the ASC instrument across the two survivor groups (long- and short-term) was provided using a multiple-group confirmatory factor analysis (CFA) with Jöreskog and Sörbom's LISREL 8.7 software [16]. See Figure 1 for the hypothesized model. This approach allowed for a comprehensive assessment of measurement invariance [17-22] across the two groups. Complete measurement invariance would imply that the two groups are identical for every aspect of cancer worry.

## Results

### Missing values

Before the analyses were performed, an examination of missing values was conducted. For the six-item ASC, the number of missing values were 30, 33, 22, 20, 15, and 140 for the items diagnostic tests, another cancer, recurrence, death, health, and children's health, respectively. The larger number of missing of values for the children's health item was a result of several individuals not having children (see Table 2). For the PANAS and CES-D instruments, there were 52 and 74 individuals, who did not respond to at least one item on the respective instruments. All subsequent analyses were performed with both listwise and pairwise deletion of missing values. However, listwise deletion was our preferred method and the pairwise deletion analyses were only performed for comparative purposes. After listwise deletion of missing values there were data from 454 short-term and 126 long-term survivors.

In order to assess whether or not the missing values were missing at random, we created six dummy variables, one for each of the six cancer worry items. If the cancer worry item was missing, then the corresponding dummy item was coded a one; if not missing, then a zero. Models were then run to assess whether there were statistically significant relations between the dummy variables and the demographic, clinical, cancer worry, PANAS, and CES-D variables.

As expected, missing values on the children's health item were related to marital status and number of children (people without partners or without children were more likely to skip the item). Additionally, people who were older, of lower income, and less educated, were more likely to skip the three cancer worry items (recurrence, new diagnosis, and diagnostic tests). This suggests that generalizations of the cancer worry subscale to older, lower income, and less educated populations should be made only with caution, and further research involving these groups is needed.

### Reliability

As assessment of the reliability (internal consistency), coefficient alpha was computed for the cancer worry and health worry subscales. Alpha was equal to 0.93 and 0.63, respectively. The values were equivalent (to two decimal places) for the listwise and pairwise deletion of missing value methods. This suggested that the cancer worry subscale had excellent reliability, but that the health worry subscale was in need of improvement.

### Check for violations of underlying assumptions

The sample was checked for multivariate outliers by calculating the Mahalanobis distance [23] (the distance from the multivariate centroid of the six items) for each person. With an α = .001 cutoff level (chosen to ensure a low risk of false outliers), no multivariate outliers were found. Multivariate normality was assessed by calculating the normalized estimate of Mardia's multivariate kurtosis coefficient [24]. This coefficient was equal to 31.7 (*p *< .0001), indicating that the variables were not distributed with multivariate normality.

Additionally, models were run to assess nonlinear relations between the six items. The square of each item was calculated and used to predict the remaining items in separate regression models. For example, in one model recurrence worry was the outcome, and the predictors were death worry and the square of death worry. The square of death worry variable indicated if there were a nonlinear relation between death worry and recurrence worry. In all models (15 total), no statistically significant nonlinear relations (*p *< .01) were found.

Because the data were ordinal and not distributed with multivariate normality, two methods were used to estimate the model parameters and fit. First, the standard method of maximum likelihood (ML) estimation [16] incorporating the covariance matrix and means from both groups was run. Second, the diagonally weighted least squares (DWLS) method with the polychoric correlation matrices [16,25] for each group was run. DWLS also required an estimate of the asymptotic covariance matrix of the sample correlations for each group. Both the polychoric correlation matrices and the asymptotic covariance matrices were calculated with Jöreskog and Sörbom's PRELIS software [26]. Although the standard ML method has been shown to be fairly robust under violation of the multivariate normality assumption, the DWLS method is preferred when violations are severe [25]. We compared the results from both methods to determine whether or not the different methods led to different conclusions.

### Goodness-of-fit indices

For assessments of model fit, we chose three goodness-of-fit indices: the root mean square error of approximation (RMSEA), the comparative fit index (CFI), and the non-normed fit index (NNFI; also called the Tucker-Lewis index, TLI) [27]. For the RMSEA, values less than .06 are considered indicative of good model fit. For the CFI and NNFI, good fit is implied by values greater than .90 [27]. We also examined χ^2 ^values for purposes of assessing improvement in model fit across nested models. When models are nested (identical in structure, but differing in the number of free parameters) the change in χ^2^, Δχ^2^, across the models provides a significance test for improvement in model fit (the degrees of freedom for the comparison is equal to the difference in degrees of freedom across the models). If a less restricted model does not show improved fit over a more restricted one, then the more parsimonious restricted model is the appropriate one. If the less restricted model does have significantly improved fit, then the restricted model must be abandoned.

The overall χ^2 ^test, which tests the null hypothesis of perfect model fit, was also reported because of its ubiquitous reporting in the confirmatory factor analytic literature. However, because of its sensitivity to sample size, this test is generally considered to be an appropriate measure only when the total sample size is less than 200 [28]. Further, this test is affected by the correlations between the variables in the model, with higher correlations suggesting poorer fit. The present analyses have a sample size greater than 200 and the correlations within the model were expected to be fairly high (because all variables were hypothesized to reflect worry). Therefore, the χ^2 ^test was not viewed as an appropriate fit index for the present study, except when comparing nested models.

### Measurement invariance

Five separate nested CFA models were run, with each subsequent model adding restrictions to the differences allowed across the two groups. The first model tested form (pattern) invariance between the two groups [17-22]. If form invariance is indicated, it implies that the hypothesized model is valid for both groups (construct validity is demonstrated for both groups), and that both groups have the same overall structure (in the path model, the same directional arrows are valid for each group). Results showed support for form invariance (see Table 5, original six-item models). All three fit indices were in the range of good fit.

**Table 5:** Maximum likelihood goodness-of-fit indices for confirmatory factor models

**Original Six-Item ASC**									
Invariance	χ^2^	*df*	*p*	Δχ^2^	*df*	*p*	RMSEA	CFI	NNFI

Form	32.0	16	.01				.055	.992	.985
Slope	33.7	22	.05	1.7	6	.95	.038	.994	.992
Intercept	39.9	28	.07	6.2	6	.40	.035	.994	.994
Item Error	46.1	34	.08	6.2	6	.40	.033	.994	.995
Covariance	46.5	35	.09	0.4	1	.53	.033	.994	.995

**Revised Five-Item ASC (children's health item excluded)**									

Invariance	χ^2^	*df*	*p*	Δχ^2^	*df*	*p*	RMSEA	CFI	NNFI

Form	11.2	8	.19				.033	.998	.996
Slope	15.8	13	.26	4.6	5	.47	.024	.999	.998
Intercept	21.4	18	.26	5.6	5	.35	.015	.999	.998
Item Error	30.5	23	.14	9.1	5	.11	.030	.996	.997
Covariance	30.9	24	.16	0.4	1	.53	.029	.997	.997

χ^2 ^is the chi-square value for the given model with degrees of freedom, *df*, and probability, *p*. Δχ^2 ^is the difference in chi-square between the given model and the previous one. RMSEA is the Root Mean Square Error of Approximation, CFI is the Comparative Fit Index, and NNFI is the Non-Normed Fit Index. Listwise deletion of missing values was used for all of the models.

In the second model, the factor loading slopes were constrained across the groups (called slope or weak factorial invariance). If this restricted model were supported, it would indicate that for both groups the influence of the latent constructs on the observed items is equivalent. The Δχ^2 ^test (see Table 5, original six-item models) indicated that the original (form invariant) model did not have statistically significantly improved fit over the slope invariant model. Therefore the slope invariant model was more appropriate.

In the third model, the factor loading slopes and intercepts were constrained across the groups (called intercept or strong factorial invariance). If this restricted model were supported, it would indicate that both groups not only have the same slopes, but also the same means on the latent constructs. The Δχ^2 ^test (see Table 5, original six-item models) indicated that the slope invariant model did not have improved model fit over the intercept (plus slope) invariant model. Therefore the intercept (plus slope) invariant model was more appropriate.

In the fourth model, in addition to constraining the factor loading slopes and intercepts, the item error variances were constrained across the groups (called item error or strict factorial invariance). If this restricted model were supported, it would indicate that both groups not only have the same slopes and intercepts, but also the same item error variances. The Δχ^2 ^test (see Table 5, original six-item models) indicated that the intercept (plus slope) invariant model did not have improved model fit over the item error invariant model. Therefore the item error invariant model was more appropriate.

Finally, in the fifth model, in addition to all the constraints in the item error invariant model, the latent covariances (the covariance between the cancer worry and health worry factors) were constrained across the groups (called latent covariance invariance). If this restricted model were supported, it would indicate that both groups not only have the same slopes, intercepts, and item error variances, but also the same latent covariances. The Δχ^2 ^test (see Table 5, original six-item models) indicated that the item error (plus slope and intercept) invariant model did not have improved model fit over the latent covariance invariant model. Therefore the latent covariance invariant model was more appropriate.

In the latent covariance invariant model, all possible parameters were constrained across the two groups. Because this model was shown to be the best one, it can be concluded that both long- and short-term survivors have identical structure and complete measurement invariance with respect to the cancer and health worry constructs (as measured by the six items developed here). See Figure 1 for the standardized parameter estimates. The correlation between the cancer and health worry constructs was .47, confirming our hypothesis that the constructs were related but distinct.

### Alternate analyses

In order to assess issues of missing values and violations of multivariate normality, we examined the results when performing the analyses using pairwise deletion of missing values, and when using the DWLS estimation method. Fortunately, when using DWLS the goodness-of-fit indices were all on the side of the threshold that indicated good model fit, indicating that the maximum likelihood method provided unbiased results. However, when pairwise deletion of missing values was used, results were not the same as with listwise deletion. Specifically, when using pairwise deletion, the three health worry items (death, health, and children's health) exhibited neither intercept invariance nor item error invariance across the long- and short-term survivor groups. The long-term survivors had a higher mean on the health worry construct than the short-term survivors did.

These differences across the listwise and pairwise deletion methods were most influenced by missing values on the children's health item. This item had substantially more missing values than the other items due to several individuals not having children. With listwise deletion, all people with no children were excluded from the analyses, but with pairwise deletion they were not. The differences in the results across the listwise and pairwise deletion methods suggest the possibility that long-term survivors with no children had higher scores on the health worry factor than short-term survivors with no children. For people with children (listwise deletion results), however, no differences across survivor groups were found. For the cancer worry factor, the results were unchanged across the listwise and pairwise deletion methods, suggesting that this construct was equivalent for both people with and without children.

Because of these issues with the children's health item, for comparative purposes, we reran each of the models with this item excluded. Missing values were handled with the listwise deletion method, and the resulting samples sizes were 546 short-term survivors and 152 long-term survivors. The conclusions were unchanged from when the item was included. Form, slope, intercept, item error, and covariance invariance were all still supported (see Table 5, revised five-item models). This suggests that a revised five-item ASC (with the children's health item excluded) is preferable to the original six-item instrument. The five-item model showed virtually the same level of construct validity (as demonstrated by the confirmatory factor models), and was free of the missing value problems resulting from the children's health item. Additionally, the coefficient alpha for the two-item health worry subscale was 0.72 (this is compared to 0.63 for the three-item subscale).

### Convergent and discriminant validity

As a further assessment of construct validity, assessments of convergent and discriminant validity were made for both the original six-item ASC and the revised five-item ASC (children's health item excluded). Correlations between the cancer and health worry subscales, and the PANAS (positive and negative subscales) and CESD were calculated. Scores for each of the subscales were computed as the mean of the set of items representing the given subscale. If a person had missing values on some, but not all, of the items for a given subscale, then the mean was calculated from the items that were available. Computed subscale scores were only missing if all of the representative items were missing. Because the short- and long-term survivor groups were found to be equivalent (complete measurement invariance), separate correlations by group were not necessary.

For the assessment of convergent validity, we set the criterion that the cancer and health worry subscales would correlate with the PANAS Negative subscale and the CES-D, and be statistically significant at the 0.001-level. For the assessment of discriminant validity, we set the criterion that the cancer and health worry subscales would *not *correlate with the PANAS Positive subscale (*not *statistically significant at the 0.001-level). The correlations between the two ASC subscales and the PANAS (positive and negative) and CES-D are shown in Table 6. Results for both the original six-item ASC and the revised five-item ASC are shown. Convergent validity was clearly demonstrated, as all were statistically significant at the .001-level, with correlations ranging from .19 (cancer worry and CES-D) to .46 (health worry and PANAS Negative). The highest value of .46 indicated that 20% of the variance is shared is between health worry and PANAS Negative. This value is not considerably high, suggesting that cancer worry and health worry are related to, but distinct from, negative affect measures.

**Table 6:** Convergent and discriminant validity correlations

**Original Six-Item ASC**								
			Cancer Worry			Health Worry		

Validity	Instrument	Subscale	*n*	*r*	*p*	*n*	*r*	*p*
Convergent	PANAS	Negative	722	.34	*	722	.46	*
	CES-D	Total	727	.19	*	725	.39	*
Discriminant	PANAS	Positive	726	.11	.003	724	.06	.10

**Revised Five-Item ASC (children's health item excluded)**								

			Cancer Worry			Health Worry		

Validity	Instrument	Subscale	*n*	*r*	*p*	*n*	*r*	*p*
Convergent	PANAS	Negative	722	.34	*	721	.43	*
	CES-D	Total	727	.19	*	724	.39	*
Discriminant	PANAS	Positive	726	.11	.003	723	.02	.67

*n *is the sample size, *r *is the correlation, and *p *is the probability value. An asterisk (*) indicates that the *p*-value is less than 0.0001. Sample sizes vary because of missing values.

Discriminant validity was also shown, especially between health worry and PANAS Positive (*r *= .02, *p *= 0.67). The correlation between cancer worry and PANAS positive was nearly statistically significant at the 0.001-level (*p *= 0.003), but the magnitude of effect was small (*r *= 0.11, only 1.2% of the variance was shared).

## Discussion

The purpose of this study was to develop a new cancer worry questionnaire, the Assessment of Survivor Concerns (or ASC). Previously, measures specific to this construct have been either included as part of a larger scale, or not vigorously tested for reliability and validity. Our objective was to keep the instrument simple and easy to use, and we therefore developed six items with three specific to cancer worry, and three applying to general health worry. Our assessments of reliability and validity for the questionnaire have shown that, in its present form, the ASC instrument is well-suited for research in which a cancer concerns measure is needed. Construct validity was demonstrated through confirmatory factor analysis and through examinations of convergent and discriminant validity. Additionally, the instrument was shown to be invariant across two important cancer survivor groups, short- and long-term survivors. This was apparent even though there were many more short-term than long-term survivors (592 versus 161). However, because of the relatively small number of long-term survivors, extra caution should be used in interpreting the long-term survivor results.

While the instrument appears solid, there are areas in need of improvement. Specifically, the health worry subscale did not fare as well as the cancer worry subscale. The health worry subscale had lower reliability and was not robust under different missing value deletion methods. An issue that might have been contributing to these problems was the children's health item. Of the six questionnaire items, children's health had the lowest factor loading and the highest number of missing values, due to the fact that several participants in the study did not have any children. Because of the problems with the children's health item, we re-ran our analyses with this item excluded, and the results showed no detrimental effect to the validity of the ASC. Hence, it is our recommendation that the revised five-item ASC be used instead of the originally proposed six-item instrument. Alternatively, if researchers do not need the health worry subscale, it can be excluded, with only the three-item cancer worry subscale used.

We should note that the results presented here might not generalize to cancer populations with older, less educated, and lower income individuals because those individuals were more likely to skip items. We should also note that the ASC does not include many other worries that cancer survivors – and particularly specific subgroups – may experience. For example, young cancer survivors may be worried about whether or not their fertility has been affected by cancer treatment. Additional questionnaires are needed for concerns other than those related to recurrence and general health.

It is interesting that our results are consistent with those of Avis, et al. [8] (which we were not aware of when conducting this study). Their questionnaire includes a four-item distress-recurrence factor comprising four items (worry about cancer coming back, worried that pain indicates cancer, worried about dying from cancer, preoccupied with cancer concerns). There are some differences in our findings–the ASC items about death and health made up a separate health worry factor, and the other three items comprised a specific cancer worry factor. However, there is specific overlap in one item and similar levels of factor loadings. Our study included a much larger sample, and a majority of short-term survivors, in contrast to Avis, et. al.'s inclusion of only long-term survivors. Thus, it is encouraging that the construct and measurement of survivor concerns is reasonably robust across investigators and survivor participants.

## Conclusion

In conclusion, we recommend the revised five-item ASC instrument when an assessment of cancer survivor concerns is needed. The ASC has excellent internal consistency and validity, and is appropriate in both short-term and long-term survivor populations.

## Competing interests

The author(s) declare that they have no competing interests.

## Authors' contributions

CG conceived of and coordinated the study, and helped to draft the manuscript. IP designed the study, performed the statistical analysis, and drafted the manuscript.
